# Sanitary Conditions of Food Vending Sites and Food Handling Practices of Street Food Vendors in Benin City, Nigeria: Implication for Food Hygiene and Safety

**DOI:** 10.1155/2014/701316

**Published:** 2014-09-03

**Authors:** P. W. Okojie, E. C. Isah

**Affiliations:** Department of Community Health, University of Benin Teaching Hospital, PMB 1111, Benin City 30001, Nigeria

## Abstract

*Objective*. To determine the sanitary conditions of vending sites as well as food handling practices of street food vendors in Benin City, Nigeria. *Methodology*. A descriptive cross-sectional study was done using an observational checklist and researcher-administered questionnaire. 286 randomly selected vending units were surveyed, and their operators interviewed on their food handling practices. *Results*. A higher proportion, 259 (90.5%), of the observed vending sites appeared clean. The following sanitary facilities were observed in and around the respective food premises of the respondents: waste bin, 124 (43.4%), refuse dumpsite, 41 (14.3%), wash hand basin, 201 (71.2%), hand towel, 210 (73.4%), and soap, 220 (76.9%). There were also the presence of flies 118, (41.3%), and the presence of rats/cockroaches, 7 (2.4%). Respondents with tertiary education, 5 (38.5%), vended foods in environment with good hygiene status compared to those with secondary, 45 (31.7%), and primary education, 33 (27.3%). There was no statistically significant association between educational status and the hygiene status of food premise (*P* = 0.362). *Conclusion*. This study showed that street food vending sites in Benin City were sanitary and that food vendors had good food handling practices.

## 1. Introduction

In many developing countries including Nigeria street food vendors have formed an integral part of the food supply chain, particularly following the advent of urbanization [1]. The street food industry has contributed immensely to human and economic development as studies conducted in some of the African countries like Nigeria, Morocco, and Kenya have shown that major street food vendors usually earn above the countries' minimum wage [2–4]. The socioeconomic role of the street food sector in terms of its potential for employment creation, yielding income particularly for women, and provision of food at affordable cost to lower income groups in the cities has been documented [5]. In Nigeria, urban city dwellers spend as much as half of their food expenditure on street foods [6].

As in other developing countries, the street food sector in Nigeria is confronted with challenges. There is inadequate supervision and proper monitoring by food safety officers and the enforcement of food hygiene regulation is weak [7]; lack of training in food safety and good hygiene practices is also rife among food handlers. Hence street foods are at risk of contamination, often at all stages of handling. Street foods are sometimes stored at improper temperatures and sold from vending sites which include kiosks, make-shift accommodation, and push carts as well as other temporary structures [8]. They are prepared at very dirty surroundings with waste water and garbage disposed nearby, providing nutrient and breeding ground for rodents and vermin [9]. In most cases running water is not available at vending sites, washing of hands and crockery are done in bowls or buckets and sometimes without soap [9, 10]. Furthermore the conditions under which street food is prepared and vended are worsened by weak implementation of relevant environmental and public health regulations.

As an integral part of its National Health Policy, Nigeria launched the National Policy on Food hygiene and Safety in 2000. Responsibilities for food safety and hygiene practice devolve on different tiers of government and their agencies—federal, state, and local. The control and regulation of street food vending, catering establishment, and the enforcement of food hygiene and safety are, however, carried out at the local government level, where Health Officers enforce relevant sections of the Public Health Laws [11]. In some cases, local government council authorities develop byelaws which apply specifically to the regulation and control of food premises. Specific laws enacted to ensure food safety in Nigeria include The Public Health Law/Ordinance Cap 164 (1917/1958), Standards Organization of Nigeria (SON) Decree (1971), the Food and Drugs Decree number 35 (1974), the Animal Disease Control Decree number 10 (1988), and the Marketing of Breast Milk Substitute Decree number 41, (1990). Others are Consumer Protection Council Decree number 66 (1992), National Agency for Food and Drugs Administration and Control (NAFDAC) Decree, number 15 (1999) and, the Counterfeit/Fake drugs/unwholesome processed Food Decree, number 15, 1999 [3]. The need to revise some existing food safety legislations has been emphasized, as some of them are out of date and do not meet current realities and trends in food safety [11].

In Nigeria, there are a large number of local eateries, where a significant number of people eat daily [12]. There has also been an observed increase in the patronage of ready-to-eat food vendors within Benin City, Edo State [12]. However, their poorly regulated operations raise serious questions about food safety and hygiene standard, as well as monitoring by relevant authority [9, 10]. Benin City is a fast growing urban centre, expanding rapidly in size and population and is characterised by “people on the move”; this creates a suitable environment for the street food trade which unfortunately operates under unsanitary conditions. These circumstances make a survey of safety and hygiene of street foods being served in the city both timely and unavoidable for early identification of emerging food safety issues in order to prevent them from developing into health risks. This study will generate information that can aid health and environment authority to improve on the implementation of existing food hygiene laws, leading to a more effective regulation of the practice of street food vending in Benin metropolis. This study was therefore undertaken to determine the sanitary conditions of vending sites as well as food handling practices by food vendors on the streets of Benin City, Nigeria.

## 2. Methodology

### 2.1. Study Area

This study was conducted in Benin City, located in the South-South geopolitical zone of Nigeria. The city is made up of three Local Government Areas, namely, Oredo, Egor, and Ikpoba-Okha. The population of Benin City based on a seven-year projection (from the 2006 census figure  1, 085676) [13] is 1,326,196. The inhabitants of Benin City are mainly small- to medium-scale business owners, farmers, artisans, civil servants, bankers, and students. The people are a combination of Christians, Muslims, and traditionalists. The majority of the inhabitants are Benin-speakers. There are major markets and abattoirs within the city that also serve as points of preparation and sale of street foods. Popularly vended street foods in Benin include local snacks like bean cake, roasted plantain, and bean pudding. Among the main meals are rice, beans, and spaghetti, amongst others.

### 2.2. Study Design

A descriptive cross-sectional study design was used in conducting this study. The study hypothesised that food safety training had a positive influence on a food vendor's practice of food and environmental hygiene. The study population comprised food vendors in their respective food vending units within the Benin City metropolis.

### 2.3. Inclusion and Exclusion Criteria

Stationary food vending units used for preparation/sales of street food as well as operators of such were included in the study. All mobile food vending units such as push carts and their vendors were excluded.

### 2.4. Study Duration

The study was carried out over a period of four months from April 2012 to July 2012 among street food vendors/vending units in the Benin metropolis.

### 2.5. Sample Size Determination

The minimum sample size of 272 for food vending sites was calculated using the formula *n* = *Z*2*pq*/*d*2 for a cross-sectional study [14].

### 2.6. Sampling Technique

A walk-through survey of randomly selected food vending sites was done and a total of 286 vending units were observed using a checklist. One vendor per vending site was selected by simple random sampling technique to participate in the survey.

### 2.7. Data Collection Tools

A structured researcher-administered questionnaire was used to collect data on background characteristics of respondents. An observational checklist adapted from the “WHO essential requirement for the safety of street-vended foods” was used to determine sanitary conditions of food vending sites/vending practices of food vendors [15]. The study instrument was pretested among selected street food vending sites/vendors operating in Ekpoma, Esan West Local Government Area of Edo State, located 50 kilometres from the study area.

### 2.8. Data Analysis

Questionnaires were screened for completeness, coded, and entered into the Statistical Package for Scientific Solution (SPSS) version 17.0 software for analysis. Discrete data were presented as proportions while continuous variables such as age were expressed as means ± standard deviation. Statistical analysis of difference between proportions was carried out by the use of *χ* ^2^ (Chi squared test). Fisher's exact test was used to test for association between variables when 20% cells or more had expected values less than five. Statistical significance was set at *P* < 0.05.

#### 2.8.1. Vending Practices

A total of ten items were assessed: (1) food sold from tray/basin with covering; (2) food sold from tray/basin with no covering; (3) food exposed to flies; (4) food reheated before sale; (5) vendor wearing hand jewellery; (6) vendor having long nails; (7) vendor having boils/cuts on the hand; (8) vendor using apron; (9) vendor having hair covering; and (10) vendor blowing air into cellophane bag before serving. A score of “1” was assigned for the correct practice while a score of “0” was assigned for wrong food handling/vending practice. The total score was converted to 100 percent. A score of ≤50% was classified as poor vending practice while a score of >50 was classified as good vending practice.

#### 2.8.2. Sanitary Condition of Vending Premises

A total of eight parameters were assessed: (1) appearance; (2) presence of waste bin; (3) presence of nearby refuse dump site; (4) presence of wash hand basin; (5) presence of hand towel; (6) presence of soap; (7) presence of flies; and (8) presence of rats/cockroaches. A score of “1” was assigned for the presence of relevant item while a score of “0” was assigned for its absence. The total score was converted to 100 percent. A score of ≤50% was classified as poor environmental hygiene while a score of >50 was classified as good environmental hygiene.

## 3. Ethical Considerations

Ethical approval was obtained from the University of Benin Teaching Hospital's Research Ethics Committee before the commencement of this study. Permission was obtained from the respective city area council authorities. Written informed consent was obtained from each respondent before the conduct of interviews after adequate information was provided by the interviewers. Confidentiality and privacy were respected during the course of the study: serial number rather than name was used to identify each respondent. Names and addresses of food premises were not used as they were rather coded. There were no penalties or loss of benefit for vendors who refused to participate in the study or withdraw from it. Group health education was conducted for food vendors who participated in the study.

## 4. Results

Two hundred and fifty-eight (90.2%) respondents were females and twenty-eight (9.8%) were males. One hundred and forty-two (49.7%) of the respondents attained secondary level of education; forty-two percent (42.3%) of them attained primary education. Twenty-eight percent of them had food safety training (Table 1).

Respondents with tertiary education, 5 (38.5%), vended foods in environment with good hygiene status compared to those with secondary, 45 (31.7%), and primary education, 33 (27.3%). There was no statistically significant association between educational status and the hygiene status of food premise (*P* = 0.362). 142 (69.6%) respondents who had no training in food safety vended foods in premises with poor environmental hygiene compared with their counterparts who had training in food safety, 56 (68.3%). There was no statistically significant association between having training in food safety and the hygiene status of food premise (*P* = 0.827). One-third (32.5%) of respondents who were not aware of food safety law/regulation vended food in good hygienic environment were compared to 25 (27.2%) of vendors who were aware of food safety law/regulation. There was no statistically significant association between awareness of food safety law and the environmental hygiene of food premise (*P* = 0.364) (Table 2).

A high proportion 259 (90.5%) of the vending sites appeared clean. The following were observed in and around the respective food premises of the respondents: waste bin, 124 (43.4%), refuse dumpsite, 41 (14.3%), wash hand basin, 201 (71.2%), hand towel, 210 (73.4%), soap, 220 (76.9%), presence of flies, 118 (41.3%), and presence of rats/cockroaches, 7 (2.4%) (Table 3).

Two hundred and seventy (94.4%) respondents were observed to vend food from containers with cover while 31 (10.8%) of them sold food to customers from containers without cover. In 79 (27.7%) of cases, vended foods were exposed to flies. Other practices observed among vendors were reheating of food, 66 (23.1%), keeping of long nails, 19 (6.6%), wearing of head cover, 208 (72.7%), and blowing of air into cellophane bags for serving food, 66 (23.1%). The majority, 237 (84%), of the vendors were observed to have good food handling practices at the point of sale. Only 49 (16%) of the vendors were observed to have poor vending/food handling practice on a cumulative scale (Table 4).

Most, 27 (96.4%), of the vendors who vended food from containers with covers were males, compared to 243 (94.2%) of their female counterparts who did. There was no significant association between gender and the practice of covering food containers (*P* = 0.624) (Table 5).

## 5. Discussion

In Africa, women are commonly involved in preparation and serving of food; they are taught how to cook from a young age. The predominance of female food vendors (90.2%) in this study is therefore not surprising. In Nigeria women are popularly involved in street food vending [2], as they depend on it as a means of complementing family income in the midst of a harsh economy. Previous studies conducted in Ilorin, Abeokuta, in Nigeria and Accra in Ghana, respectively, showed similar trend [2, 16, 17]. This was, however, not the case in studies conducted in South Africa and Kenya, respectively, where street food vendors were mostly males [18, 19]. Gender role in socioeconomic development is often influenced by cultural orientation and varies from one geographical location to another.

There were more respondents who had attained secondary education. A study among two hundred street food vendors in South Africa documented a similar finding, where the majority, 181 (90.5%), of the study participants attained secondary education [20]. However, it contrasts with other studies in which most street food vendors had only attained primary education [21, 22]. This may be attributed to the fact that most street vendors belong to the socioeconomically disadvantaged group and hence may lack a ready opportunity to pursue higher education due to social deprivation occasioned by poverty. In addition, many cultures in Africa invest less in female education, making the girl child socially disadvantaged in a male-dominated world.

Food hygiene and safety training among a small proportion of the respondents as observed in this study is similar to findings from a study conducted among 185 food vendors in secondary schools in Ilorin, Nigeria, in which only 39.0% of the respondents had a form of training in food hygiene and safety [23]. This activity may not be regularly organized in the study area by the concerned authority. Apathy towards food safety seminars can also be a reason particularly when food vendors rely more on their experience from long years of practice [19]. The public health laws prescribe that health officers organize regular trainings and food safety education for food handlers. Some unwholesome food handling practices of food vendors are deeply rooted in traditions and customs and thus require messages tailored to address such beliefs.

The observation of good sanitary condition in the majority of the food vending sites is similar to findings of studies conducted in Owerri, Nigeria, and Accra, Ghana [16, 24], where the majority of the food premises were observed to be tidy, with the use of waste bin and the presence of on-site water source for sanitary purposes. This phenomenon is in line with requirements of standard guidelines and recommendations for street food vending practice and is thus commendable [15, 25]. This finding is, however, at variance with that of a study in Brazil where the sanitary conditions of sale points of commercial foods of plant origin vended in Sao Gancalo and Rio de Janeiro were poor [26]. Not only are sanitary conditions in and around an eatery suggestive of health consciousness by the service provider, they are also likely to boost consumer confidence and increase vendor patronage.

Vendors who had no formal training in food hygiene and safety appeared to vend food in premises with poor environmental hygiene compared with their counterparts who had food safety training though the statistical test did not support its invariability. By implication it can be opined that receiving food safety training may not always lead to maintaining good environmental hygiene on the part of the vendors. Among other factors, a positive attitude is required on the part of the vendor to translate the knowledge gained from trainings into practice. This is not to say however that food safety training is less contributory to food hygiene. There was no statistically significant association between awareness of food safety regulation and the hygienic status of the food premises even though the majority of respondents who vended in premises with good environmental hygiene were aware of food safety regulation. By implication, awareness of food safety regulation did not necessarily influence good environmental hygiene among the respondents. However, more public health enlightenment is required to sensitize food vendors on the need to maintain environmental sanitation as unsanitary environment and other nuisance gives opportunity for breeding of insect/vectors, including vermin that can compromise food product quality. According to the public health laws, as part of their food safety enforcement roles, health officers are empowered to abate such nuisances in a food premise and destroy any food material or product which constitutes a food safety risk.

Most of the respondents vended food items in environments that were observed to be clean. This finding is, however, at variance with what was reported in a Kenyan study where it was observed that about 85.0% of the vendors prepared their food in unhygienic condition [19]. A clean food stand or premise will attract more consumers based on aesthetic appeal. Though food aesthetics is desirable, as it contributes to increase in sales, it does not rule out the significance of other aspects of food hygiene such as hand washing and regular medical examination. The practice of the use of head covers and the wearing of aprons by the vendors is commendable as many food safety regulations including the Public Health Laws of Edo State specify the wearing of caps/aprons as a part of a vendor's food safety responsibility. However, the wearing of clean-looking aprons may be contributing more to fueling consumer's subjective assessment of a vendor's sense of personal hygiene. Food vendors should not only wear clean aprons as part of food aesthetics, but they should also perform periodic medical examination and store food items in such a way that they are free of contamination.

Even though some of the vending sites had the presence of flies, the situation was better when compared with those of sanitary conditions in a study conducted in Nairobi, Kenya, where flies were present in all stalls operated by vendors of fish and fresh fruit salad [19]. Flies are mechanical vectors and thus can spread infective agents to food and water for human consumption, leading to potential foodborne diseases such as cholera, dysentery, and typhoid. This study revealed no statistically significant association between sex and how food containers were kept. Even though a higher proportion of male vendors were observed to cover their food containers most of the time compared to their female counterparts, it was not enough to conclude that male vendors had better food vending practices. In addition upbringing and training can also play a role; a girl child who is untrained in the etiquettes of handling and maintaining utensils may perform averagely below her male counterpart who had learned these skills. Thus emphasis should be placed on training for all food handlers irrespective of gender to influence their hygiene practices in the interest of public health.

## 6. Conclusion

It was found from this study that street foods were vended under relatively good sanitary conditions and that the vendors had good food handling practices. It is recommended that food vendors should continue to maintain standard environmental and personal hygiene while preparing and packaging or serving food products to consumers.

## Figures and Tables

**Table 1:** Background characteristics of respondents.

Background characteristics	Frequency (*n* = 286)	Percent (%)
Sex		
Male	28	9.8
Female	258	90.2
Educational attainment		
None	9	3.2
Primary	121	42.3
Secondary	142	49.7
Tertiary	13	4.5
Vocational	1	0.3
Food safety training		
Trained	82	28.7
Not trained	204	71.3
Awareness about food safety law		
Aware	92	32.2
Not aware	194	67.8

**Table 2:** Background characteristics of respondents and sanitary conditions of food vending sites.

Background characteristics	Environmental hygiene		
	Poor (%)	Good (%)	Total (%)
Educational attainment			
None	5 (55.6)	4 (44.4)	9 (100.0)
Primary	88 (72.7)	33 (27.3)	121 (100.0)
Secondary	97 (68.3)	45 (31.7)	142 (100.0)
Tertiary	8 (61.5)	5 (38.5)	13 (100.0)
Vocational	0 (0.0)	1 (100.0)	1 (100.0)
Total	**198 (69.2)**	**88 (30.8)**	**286 (100.0)**
Fisher's exact = 4.15		*P* = 0.362	
Food safety training			
Trained	56 (68.3)	26 (31.7)	82 (100.0)
Not trained	142 (69.6)	62 (30.4)	204 (100.0)
Total	**198 (69.2)**	**88 (30.8)**	**286 (100.0)**
*χ* ^2^ = 0.047		*P* = 0.827	
Awareness of food safety law			
Aware	67 (72.8)	25 (27.2)	92 (100.0)
Not aware	131 (67.5)	63 (32.5)	194 (100.0)
Total	**198 (69.2)**	**88 (30.8)**	**286 (100.0)**
*χ* ^2^ = 0.823		*P* = 0.364	

**Table 3:** Environmental hygiene of food vending premises (*n* = 286).

	Frequency∗	Percent (%)
Environmental hygiene parameters		
Clean environment	259	90.5
Waste bin present	124	43.4
Refuse site present	41	14.3
Wash basin present	201	71.2
Presence of hand towel	210	73.4
Soap present	220	76.9
Presence of flies	118	41.3
Presence of rats/cockroaches	7	2.4
Indices of environmental hygiene status		
Good	198	69.2
Poor	88	30.8

*Multiple responses.

**Table 4:** Observed food handling/vending practices (*n* = 286).

	Frequency∗	Percent (%)
Observed practices		
Food in covered container	270	94.4
Food in uncovered container	31	10.8
Food exposed to flies	79	27.7
Food reheated before sale	66	23.1
Vendor wore hand jewelry	25	8.8
Vendor had long nails	19	6.6
Vendor had hair covering	277	98.6
Vendor had cut on the hand	78	27.3
Vendor wore apron	242	84.6
Vendor blew air into cellophane bag use for vending food	66	23.1
Indices of food handling practice		
Good	237	84.0
Poor	49	16.0

*Multiple responses.

**Table 5:** Sex and mode of keeping food containers by respondents.

	Covering of food containers		Total (%)
	Yes (%)	No (%)	
Sex			
Male	27 (96.4)	1 (3.6)	28 (100.0)
Female	243 (94.2)	15 (5.8)	258 (100.0)
Total	**270 (94.4)**	**16 (5.6)**	**286 (100.0)**
*χ* ^2^ = 0.24		*P* = 0.624	

## References

[1] Akintaro OA (2012). Food handling, hygiene and the role of food regulatory agencies in promoting good health and development in Nigeria. *International Journal of Health and Medical Information*.

[2] Omemu AM, Aderoju ST (2008). Food safety knowledge and practices of street food vendors in the city of Abeokuta, Nigeria. *Food Control*.

[3] Ifenkwe GE (2012). Food safety regulation: reducing the risk o f food-borne diseases in rural communities in Abia state, Nigeria. *Agricultural Science Research Journal*.

[4] Mwangi A (2002). *Nutritional, hygienic and socioeconomic dimension of street foods in urban areas: the case of Nairobi [Ph.D. thesis]*.

[5] Tavonga N (2014). Operations of street food vendors and their impact on sustainable urban life in high density suburbs of Harare, in Zimbabwe. *Asian Journal of Economic Modelling*.

[6] Cohen MJ, Garnet JL (2002). The food price crisis and urban food (in)security. *Human Settlement Working Paper Series; Urbanization & Emerging Population no.*.

[7] Oyeneho SN, Hedberg CW (2013). An assessment of food safety needs of restaurants in Owerri, Imo State, Nigeria. *International Journal of Environmental Research and Public Health*.

[8] Food and Agricultural Organization (1990). Report of an FAO expert consultation. *FAO Food and Nutrition Paper*.

[9] Barro N, Bello AR, Salvadogo A, Ouattara CA, IIboudu AJ, Traore AS (2006). Hygienic status assessement of dish washing waters, utensils, hands and pieces of monies from street food processing sites in Ouagadougou (Burkina Faso). *African Journal of Biotechnology*.

[10] Abdalla MA, Suleiman SE, Alien YYHA, Bakheit O (2008). Food safety knowledge and practices of street food vendors in Khartoum City. *Sudanese Journal of Veterinary Science & Animal Husbandry*.

[11] Omotayo RK, Denloye SA (2002). *The Nigerian Experience on Food Safety Regulation*.

[12] Wogu MD, Omoruyi MI, Odeh HO (2011). Microbial load in ready-to-eat rice sold in Benin City. * Journal of Microbiology and Antimicrobials*.

[13] Federal Government of Nigeria (2007). Legal Notice on Publication of the details of the breakdown of the National and State Provisional Totals 2006 Census. *Federal Republic of Nigeria Official Gazette*.

[14] Araoye MO (2004). *Research Methodology with Statistics for Health and Social Sciences*.

[15] World Health Organization Essential safety requirement for street vended foods. http://apps.who.int/iris/handle/10665/63265?mode=full.

[16] Chukuezi CO (2010). Food safety knowledge and practices of street food vendors in Owerri, Nigeria. *Studies in Sociology of Science*.

[17] Donkor ES, Kayang BB, Quaye J, Akyeh ML (2009). Application of the WHO keys of safer food to improve food handling practices of food vendors in a poor resource community in Ghana. *International Journal of Environmental Research and Public Health*.

[18] Duse AG, da Silva MP, Zietsman I (2003). Coping with hygiene in South Africa, a water scarce country. *International Journal of Environmental Health Research*.

[19] Muindi OK, Kuri E (2005). Hygiene and sanitary practices of vendors of street foods in Nairobi, Kenya. *African Journal of Food & Nutritional Development*.

[20] Martins JH (2006). Socio-economic and hygiene features of street food vending in Gauteng. *South African Journal of Clinical Nutrition*.

[21] Georges C FAO technical support for improvement within the street food sector.

[22] Food and Agricultural Organization (1990). Report of an FAO expert consultation. *FAO Nutrition Paper*.

[23] Musa OI, Akande TM (2003). Food hygiene practices of food vendors in secondary schools in Ilorin. *The Nigerian Postgraduate Medical Journal*.

[24] Mensah P, Yeboah-Manu D, Owusu-Darko K, Ablordey A (2002). Street foods in Accra, Ghana: how safe are they?. *Bulletin of the World Health Organization*.

[26] Nunes BN, Cruz AG, Faria JAF, Sant’ Ana AS, Silva R, Moura MRL (2010). A survey on the sanitary condition of commercial foods of plant origin sold in Brazil. *Food Control*.

